# Cardiovascular Responses to Simultaneous Diving and Muscle Metaboreflex Activation

**DOI:** 10.3389/fphys.2021.730983

**Published:** 2021-10-22

**Authors:** Annalisa Di Giacomo, Giovanna Maria Ghiani, Francesco Todde, Filippo Tocco

**Affiliations:** Department of Medical Sciences and Public Health, School of Sport Medicine, University of Cagliari, Cagliari, Italy

**Keywords:** diving reflex, metaboreflex, heart rate, stroke volume, face immersion

## Abstract

**Background:** The aim of study was to assess hemodynamic changes during the simultaneous activation of muscle metaboreflex (MM) and diving reflex (DR) in a laboratory setting. We hypothesized that as long as the exercise intensity is mild DR can overwhelm the MM.

**Methods:** Ten trained divers underwent all four phases (randomly assigned) of the following protocol. (A) Postexercise muscle ischemia session (PEMI): 3 min of resting followed by 3 min of handgrip at 30% of maximum force, followed immediately by 3 min of PEMI on the same arm induced by inflating a sphygmomanometer. Three minutes of recovery was further allowed after the cuff was deflated for a total of 6 min of recovery. (B) Control exercise recovery session: the same rest-exercise protocol used for A followed by 6 min of recovery without inflation. (C) DR session: the same rest-exercise protocol used for A followed by 1 min of breath-hold (BH) with face immersion in cold water. (D) PEMI-DR session: the same protocol used for A with 60 s of BH with face immersion in cold water during the first minute of PEMI. Stroke volume (SV), heart rate (HR), and cardiac output (CO) were collected by means of an impedance method.

**Results:** At the end of apnea, HR was decreased in condition C and D with respect to A (−40.8 and −40.3%, respectively vs. −9.1%; *p* < 0.05). Since SV increase was less pronounced at the same time point (C = +32.4 and D = +21.7% vs. A = +6.0; *p* < 0.05), CO significantly decreased during C and D with respect to A (−23 and −29.0 vs. −1.4%, respectively; *p* < 0.05).

**Conclusion:** Results addressed the hypothesis that DR overcame the MM in our setting.

## Introduction

The human diving response (DR) is characterized by a hemodynamic remodeling where sympathetic and parasympathetic components of the nervous system simultaneously work to evoke bradycardia, reduced cardiac output, vasoconstriction of selected vascular beds, and increased arterial pressure. The outcome resulting is an oxygen sparing effect (Andersson et al., 2004). Conversely exercise is known to increase arterial pressure, heart rate, myocardial contractility, and ventilation. The type of exercise performed has important effects on how these autonomic effects are expressed (Kaufman and Hayes, 2002). A previous research, which investigated hemodynamic changes during simulated dynamic apnea, found a particular cardiovascular response in the second phase of dynamic apnea when a delayed increase in myocardial performance and stroke volume (SV) occurred and obscured the cardiovascular effects of diving reflex (Tocco et al., 2012). The authors of the aforementioned article hypothesized that muscle metaboreflex (MM) may act in opposition to the DR to maintain cardiovascular homeostasis. In fact, even though a subject is specifically trained, during prolonged dynamic apnea the peripheral accumulation of metabolic end-products could cause a stimulation of group III and IV nerve-endings, thereby recruiting the MM, which is known to be able to improve myocardial performance (Crisafulli et al., 2003, 2006). However, in the study by Tocco et al. (2012), the MM interference on diving reflex was only theoretical by authors since the experimental condition did not allow to isolate and to measure its hemodynamic effect. Thus, we conceived the idea to use a protocol able to evoke MM by trapping muscle metabolites in the exercising limb and maintaining stimulation of the metaboreceptors, previously tested by Crisafulli et al. (2003), along with an apnea with face immersion that evoked the diving reflex. We hypothesized that as long as the exercise intensity is mild DR can overwhelm the MM. The aim of the present study was to assess, for the first time, hemodynamic changes during isolated and simultaneous activation of MM and DR in a laboratory setting.

## Materials and Methods

### Participants

The study was conducted on 10 trained instructors of diving (six men and four women). At the time of tests, none of the female divers were in their menses phase. Information on the study procedures was provided to all participants and the study was approved by the Cagliari University Ethics Committee and conducted according to the Declaration of Helsinki. Written informed consent was obtained before subjects entered the study.

### Experimental Design

**Figure 1:**
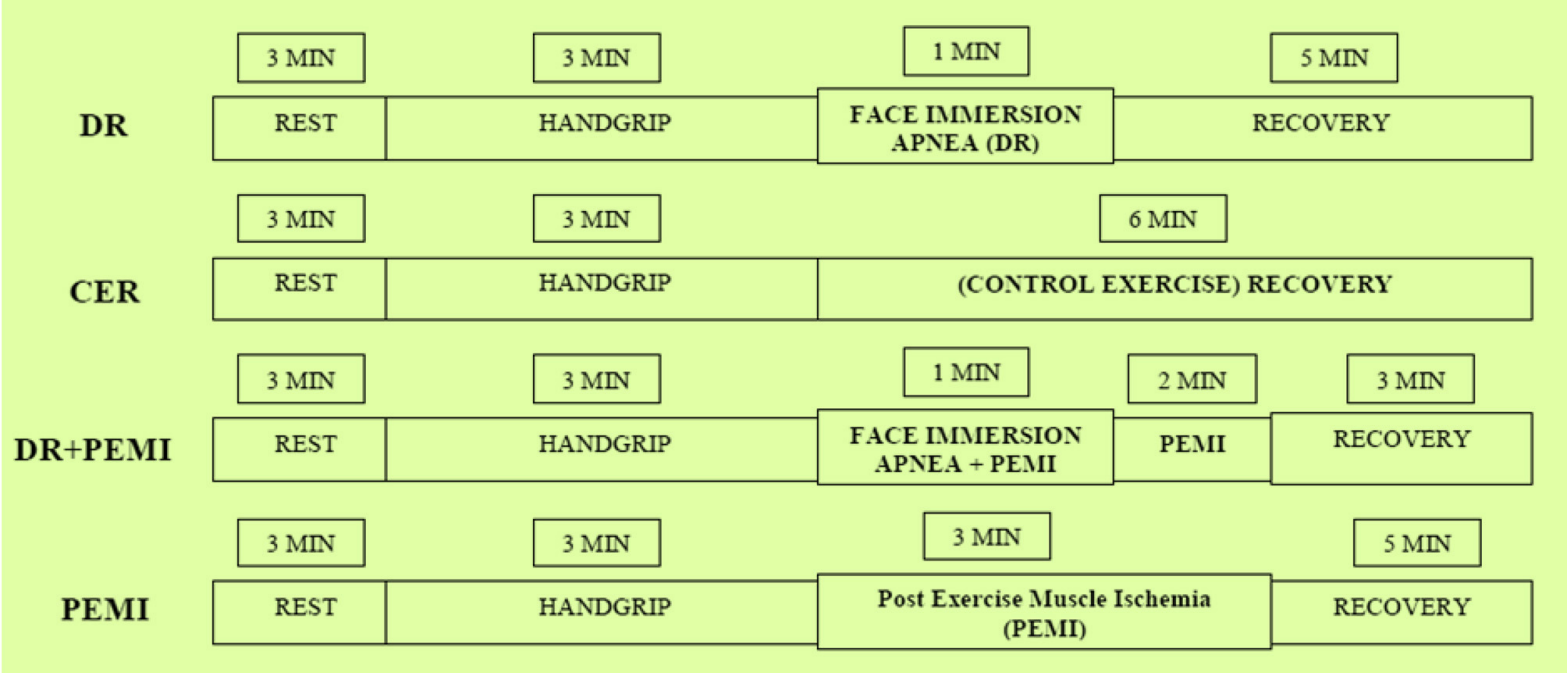
Schematic representation of the four phases of the experimental design. See text for details.

Hemodynamic, respiratory, and metabolic changes were assessed in a temperature-controlled, air-conditioned room (22°C, relative humidity 50%) at the same time in the morning. Upon arrival of subjects in the room, basic anthropometric parameters were collected for each diver (Table 1). After instrumentation, the divers adopted the sitting posture. Five minutes were allowed to achieve steady-state conditions for cardio-respiratory and metabolic parameters, and then the subjects underwent all the four phases of the following protocol randomly assigned to eliminate any order effect (Figure 1).

**Table 1:** Anthropometric parameters of divers involved in the study.

**Age (years)**	**BM (kg)**	**Height (cm)**
43.6 ± 10.1	68.1 ± 9.4	172.0 ± 7.5

*BM, body mass*.

#### Postexercise Muscle Ischemia Session (PEMI)

Divers observed 3 min of resting, followed by 3 min of mild exercise, consisting of handgrip at 30% of maximum force, followed by 3 min of PEMI on the left arm induced by rapidly inflating a tourniquet to 50 mmHg above systolic blood pressure. Three minutes of recovery was further allowed after the cuff was deflated, for a total of 6 min of exercise recovery. As stated before, this protocol has been used in several previous investigations and it has been demonstrated to be capable of trapping the muscle metabolites in the exercising limb and of maintaining stimulation of the metaboreceptors (Crisafulli et al., 2003, 2006).

#### Control Exercise Recovery Session (CER)

The same rest–exercise protocol used for PEMI followed by a control exercise recovery of 6 min without tourniquet inflation.

#### Diving Reflex Session (DR)

After the same rest–exercise protocol each diver performed 60 s of breath-hold with face immersion in cold water (water temperature 17°C). Previous investigations have demonstrated that apnea with face-immersion is capable of evoking the typical diving response (Andersson et al., 2004; Tocco et al., 2012).

#### PEMI+Diving Reflex Session (PEMI-DR)

During the first minute of PEMI divers performed 60 s of breath-hold with face immersion in cold water. Sessions were spaced by a 30-min interval during which the subject rested in order to recover completely.

### Measurements

During all the protocol phases, cardiodynamic variables were measured by means of impedance cardiography (IC) (NCCOM 3, BoMed Inc., Irvine CA). The impedance method provides noninvasive reliable data of thoracic fluid index (TFI), left ventricular ejection time (VET), SV, heart rate (HR), and cardiac output (CO). IC has been commonly employed in resting and exercising subjects (Concu and Marcello, 1993; Crisafulli et al., 2003). Impedance and ECG recorded traces were analyzed with a digital chart recorder (PowerLab 8sp, ADInstruments, Castle Hill, Australia). The SV-to-VET ratio was also assessed and considered as an index of myocardial performance (Tanaka et al., 1986; Concu and Marcello, 1993). Systemic vascular resistance (SVR) was obtained by dividing mean blood pressure (MBP, calculated as diastolic blood pressure + 1/3 systolic blood pressure – diastolic blood pressure) by CO. Systolic and diastolic blood pressure measurements were performed every 30 s. Measurements were always taken in the morning at least 2 h after light breakfast.

### Data Analysis

Since divers showed a wide dispersion in hemodynamic values at the end of exercise phase, we chose to report data as mean ± SD percent changes from end exercise level. Data were averaged for 1 min, except for the breath-hold phases, where variables were averaged for 20 s. Thus, during each trial we recorded nine time points: one for end exercise (EXE 3, which was taken as the last minute of exercise), three for the breath-hold phase (20, 40, and 60 s), and five for the recovery phase (REC 2 to REC 6, averaged for 1 min). Differences between conditions were studied by means of the two-way analysis of variance (ANOVA) for repeated measures (factors: condition and time). Tukey's *post hoc* was performed when appropriate. The level of significance was set at *P* < 0.05 in all cases.

## Results

**Figure 2:**
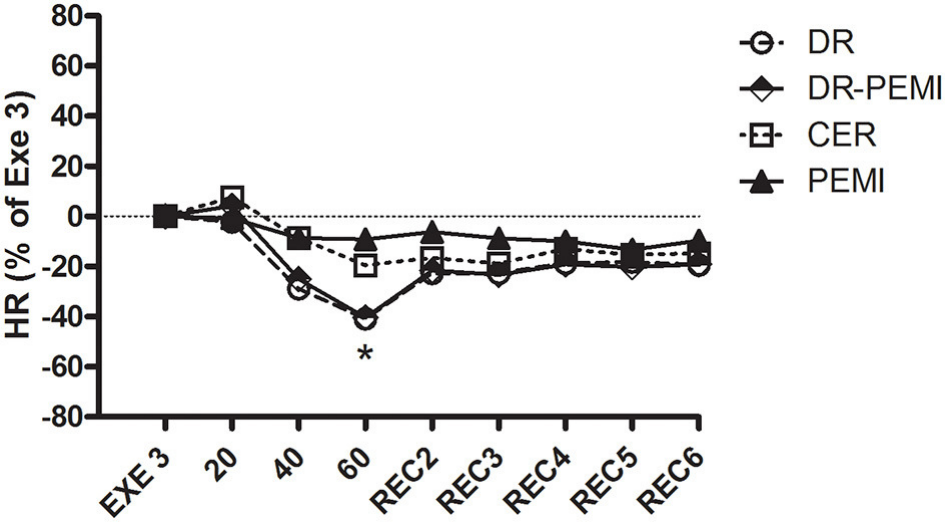
HR time courses during all four tests. Values are means ± SD percentages of the end of handgrip (EXE3). * = *P* < 0.05 vs. EXE3.

**Figure 3:**
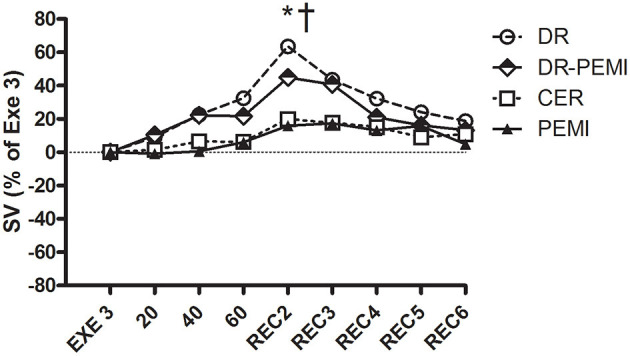
SV time courses during all four tests. Values are means ± SD percentages of the end of handgrip (EXE3). * = *P* < 0.05 vs. EXE3. † = *P* < 0.05 vs. PEMI.

**Figure 4:**
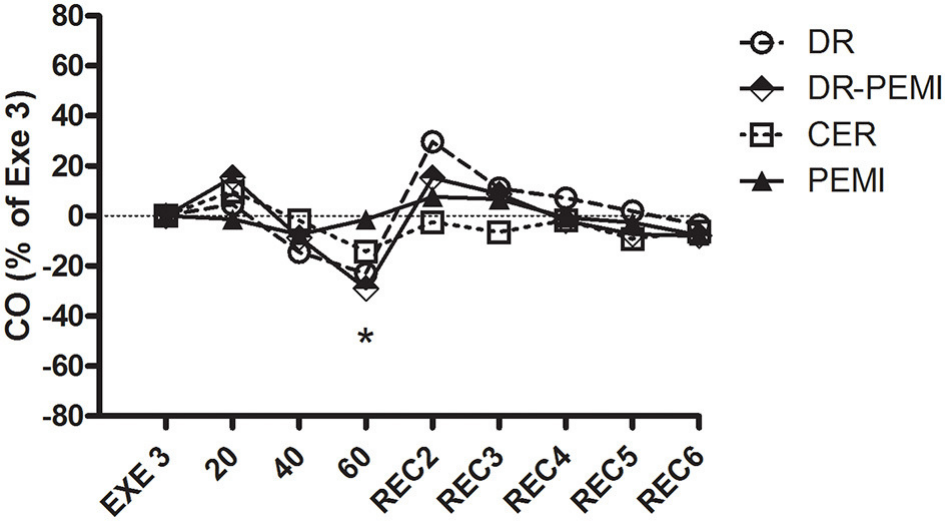
CO time courses during all four tests. Values are means ± SD percentages at the end of handgrip (EXE3). * = *P* < 0.05 vs. EXE3.

**Figure 5:**
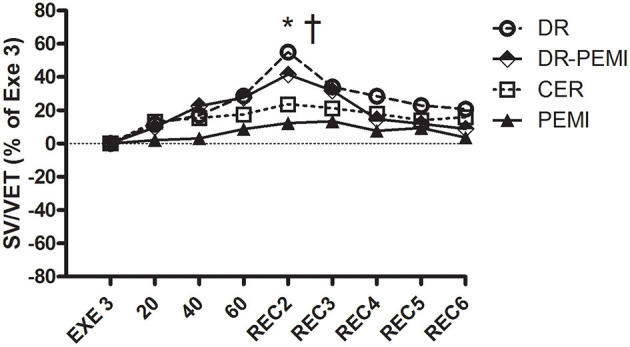
SV/VET time courses during all four tests. Values are means ± SD percentages of the end of handgrip (EXE3). * = *P* < 0.05 vs. EXE3. † = *P* < 0.05 vs. PEMI.

**Figure 6:**
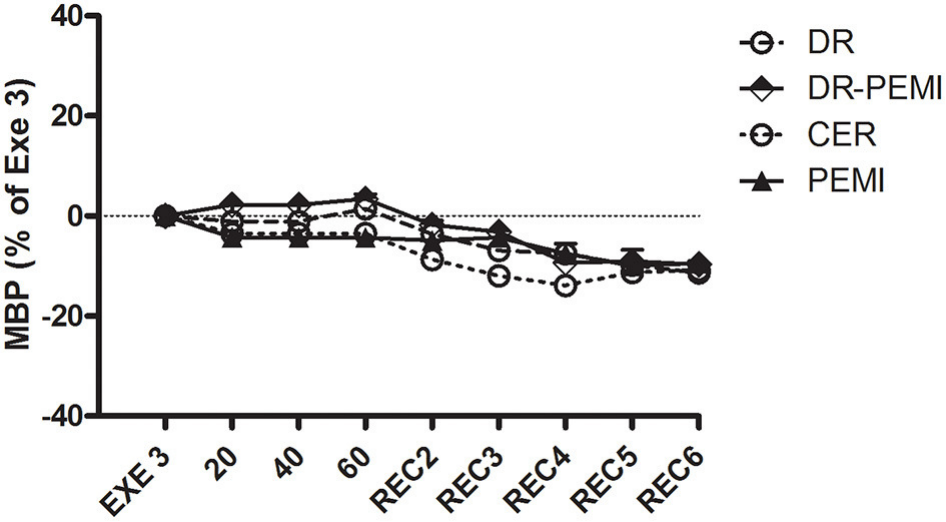
MBP time courses during all four tests. Values are means ± SD percentages of the end of handgrip (EXE3).

**Figure 7:**
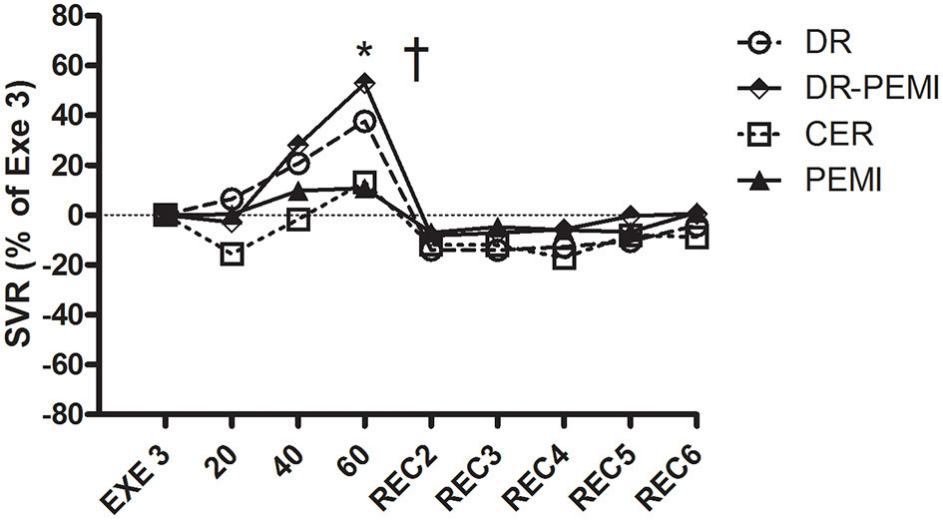
SVR time courses during all four tests. Values are means ± SD percentages of the end of handgrip (EXE3). * = *P* < 0.05 vs. EXE3. † = *P* < 0.05 vs. PEMI.

**Figure 8:**
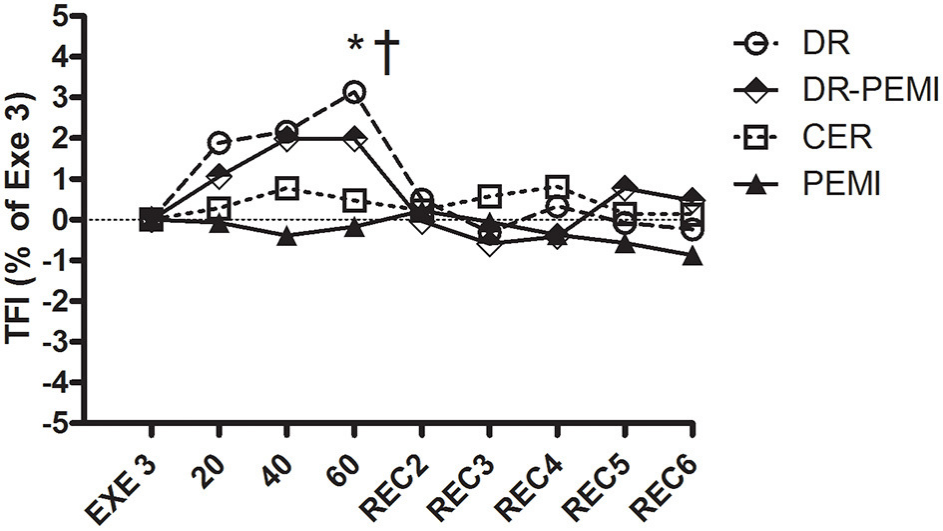
TFI Values are means ± SD percentages of the end of handgrip (EXE3). * = *P* < 0.05 vs. EXE3. † = *P* < 0.05 vs. PEMI.

The protocol was completed by all divers, and no symptoms were reported. The anthropometric values of the divers are shown in Table 1. Baseline absolute values of hemodynamic parameters are shown in Table 2. Figures 2–8 show the time courses of each cardiovascular parameter measured in divers during trials.

**Table 2:** Absolute hemodynamic and metabolic data in divers at rest preceding the apnoeas.

**Parameter (Units)**	**HR (bpm)**	**SV (ml)**	**CO (L·min^**−1**^)**	**SV/VET (ml·sec^**−1**^)**	**SVR (dyne·s/cm^**5**^)**	**MBP (mmHg)**	**TFI (Ohm)**
**Value**	72.8 ± 0.6.1	56.1 ± 14.4	3.9 ± 0.8	245.3 ± 31.2	1773.7 ± 268.8	81.6 ± 8.2	33.9 ± 1.5

*HR, heart rate; SV, stroke volume; CO, cardiac output; SV/VET, stroke volume/ventricular ejection time; SVR, systemic vascular resistance; MBP, mean blood pressure; TFI, trans-thoracic fluid index. Values are mean ± SD*.

Figure 2 shows that during DR and DR-PEMI trials, HR significantly decreased with respect to Exe 3 at time point 60. Figure 3 demonstrates that SV tended to increase in response to both DR and DR-PEMI maneuvers with respect to Exe 3. Moreover, at time point REC2 of test DR SV significantly increased with respect to PEMI. As a consequence of HR and SV behaviors, CO decreased at time point 60 with respect to Exe3 during tests DR and DR-PEMI. It increased at REC2 especially during DR trial, and then returned to reference values in the following periods (Figure 4). The SV/VET ratio (index of myocardial performance, Figure 5) gradually increased during the apnea and REC2 periods of both test DR and DR-PEMI compared with Exe3, and it was significantly higher in DR than in PEMI trial at time point REC2 (P = 0.007). This fact meant that myocardial performance was improved by the apnea maneuvers during recovery. Then, in both trials DR and DR-PEMI, SV/VET gradually returned to the Exe3 level.

Despite aforementioned hemodynamic changes during all conditions, there was no detectable divergence of MBP values (Figure 6). In DR and DR-PEMI trials, apnea induced a rise in SVR compared with EXE 3, and these differences became significant at time point 60 versus PEMI trial (Figure 7). Finally, TFI increased (i.e. thoracic fluids decreased) during the apnea phases of tests DR and DR-PEMI, and this gain was significantly higher in DR compared with PEMI at time point 60 (Figure 8). In summary, the main differences between trials detected in our study were that: 1. apnea led to bradycardia in both DR and DR-PEMI trials; 2. there was a SV delayed increment in response to apnea in both DR and DR-PEMI trials; 3. in the same trials the SV increment did not compensate for the bradycardia during apnea and, as a consequence, CO was reduced; 4. SVR augmented during apnea in DR and DR-PEMI, thus MPB was maintained.

## Discussion

Our goal it was to investigate the hemodynamic changes evoked by a simultaneous condition of apnea and mild exercise in a laboratory simulation. It is plausible that during various diving disciplines (constant weight diving, variable weight, dynamic apnea, etc.) both mechanisms (apnea and exercise reflexes) are activated. Indeed, Ichinose et al. (2018) recently demonstrated that voluntary apnea during dynamic exercise activates the MM in humans. Thus our hypothesis was that during a light intensity exercise (as happens in some diving disciplines) the diving reflex, despite metaboreflexes, could lead to cardiac output decrement and therefore an oxygen sparing effect. From the overall analysis of our results, it would seem that this is indeed the case. DR prevailed over MM in our simulation. This outcome is quite different from findings of Tocco et al. (2012) which found a particular cardiovascular response in the second phase of simulated dynamic apnoeas when a delayed increase in myocardial performance and SV occurred and obscured the cardiovascular effects of the DR. This opposite outcome could be due to the different protocol used. In fact, in the aforementioned research (Tocco et al., 2012) the divers performed a continuous dynamic exercise pedaling on a cycle-ergometer against a workload of 0.5 W kg^−1^ of body mass. At that exercise intensity level, an increase in myocardial contractility was already evident during the last part of apnea, with a consequent increase in SV. In the current research divers carried out 3 min of mild exercise, consisting of handgrip at 30% of maximum force, and there was instead an increase in contractility (SV/VET) and consequently in SV and CO immediately after apnea was stopped (REC2).

The prevalence of the apnea reflex over the exercise response is a debated topic among researchers. Delahoche et al. (2005) highlighted great importance of adequate apnea training in enhancing oxygen-saving capacity. During underwater dynamic apnea conditions, Joulia et al. (2009) found that elite divers presented a potentiating of the apnea response, whereas Tocco et al. (2013) concluded that sympathetic activation induced by exercise partially obscured the effects of the diving response. During a real free-diving in the sea, Lemaître et al. (2013) reported that the diving response was strong enough to override the stimulus of muscular exercise in elite divers that showed bradycardia performing deep dives. Different outcome was shown by Marongiu et al. (2015), which found that exercise performed during free-diving counteracted the cardiovascular effects of the diving response to ensure adequate CO toward exercising muscles.

Nutrition and dietary supplementation also appear to influence the response to diving. Engan et al. (2012) suggested that acute dietary ${\text{NO}}_{3}^{-}$ supplementation improved static apnea performance by reducing metabolic costs. Patrician and Schagatay (2017) found an oxygen conserving effect of dietary nitrate supplementation during dynamic maximal apnea performance. Ghiani et al. (2016), during real diving in the sea, reported that divers with an adequate balance between metabolic and splancnic status (i.e. during a fasting by 12 hours) improved their diving response.

The present research emphasizes the importance of exercise intensity as more or less influencing the DR. This fact has important practical implications for divers. Maximizing movements required for dynamic apnea or constant weight immersion, keeping the exercise of light intensity, would allow the DR to prevail over the response to exercise better. In our opinion, an opinion supported by the results of the present and previous researches, this is an aspect that can tip the balance between two reflexes toward the DR. Divers should focus the most of their training on the diving propulsive and sliding technique, optimizing it as much as possible. This would also improve their safety when diving.

## Limitations on the Study

The experimental design allowed us to study the diving reflex and exercise pressor response and their interactions simultaneously, but the results must be considered limited to our experimental context. In fact, for practical needs, it lacked some components of immersion such as especially the blood shift, linked to the increase in atmospheric pressure. Previous studies conducted under real diving conditions have shown how this component can modify hemodynamics compared with a laboratory situation (Joulia et al., 2009; Lemaître et al., 2013; Marongiu et al., 2015). Another possible limitation of the present investigation was the low number of subjects enrolled. However, we chose the use of percent changes instead of absolute values to describe time courses of variables, and this procedure allowed to curtail quantitative difference among subjects.

## Data Availability Statement

The original contributions presented in the study are included in the article, further inquiries can be directed to the corresponding author.

## Ethics Statement

The studies involving human participants were reviewed and approved by Cagliari State University Ethics Committee. The patients/participants provided their written informed consent to participate in this study.

## Author Contributions

AD, GG, FrT, and FiT contributed to the data analysis and interpretation of the data, drafting, and revising the manuscript, and approved the final version of the manuscript. The original study design was made by FiT and discussed with the other authors. All authors contributed to the article and approved the submitted version.

## Conflict of Interest

The authors declare that the research was conducted in the absence of any commercial or financial relationships that could be construed as a potential conflict of interest.

## Publisher's Note

All claims expressed in this article are solely those of the authors and do not necessarily represent those of their affiliated organizations, or those of the publisher, the editors and the reviewers. Any product that may be evaluated in this article, or claim that may be made by its manufacturer, is not guaranteed or endorsed by the publisher.
